# Nurses’ knowledge of stroke-related oropharyngeal dysphagia in the Eastern Cape, South Africa

**DOI:** 10.4102/sajcd.v67i1.703

**Published:** 2020-09-02

**Authors:** Kerry Knight, Bhavani Pillay, Jeannie van der Linde, Esedra Krüger

**Affiliations:** 1 Department of Speech-Language Pathology and Audiology, Faculty of Humanities, University of Pretoria, Pretoria, South Africa

**Keywords:** oropharyngeal dysphagia, dysphagia screening, stroke-related dysphagia, nurse, interdisciplinary collaboration, South Africa, lower middle-income country, survey

## Abstract

**Background:**

Early identification of stroke-related oropharyngeal dysphagia (OPD) using screening by nurses can prevent adverse patient outcomes in lower middle-income countries. Nurses are essential in the OPD management team and should ideally be able to screen and prioritise dysphagia management in stroke patients.

**Objective:**

The aim of this research was to describe nurses’ practices related to identification and management of patients with stroke-related OPD.

**Methods:**

Qualified nurses from various healthcare levels in the Eastern Cape, South Africa were invited to complete a previously published hard copy survey on the signs and symptoms, complications and management of stroke-related OPD. A sample of 130 participants completed the survey.

**Results:**

The mean scores of correct responses for each section were: 8.7/13 (66.7%) for signs and symptoms, 4.7/10 (47.3%) for complications and 3.8/7 (54.2%) for management practices. Statistically, there were no differences between the levels of healthcare for the signs and symptoms section and the complications section. Regarding management of OPD, secondary-level (S) nurses demonstrated significantly better knowledge than primary-level (P) and tertiary-level (T) nurses (S–P: p = 0.022; S–T: p = 0.010). Secondary-level nurses also scored significantly higher across all three sections (S–P: *p* = 0.044; S–T: *p* = 0.025) than those at the other levels.

**Conclusions:**

The study found that nurses across all levels of healthcare had only moderate knowledge regarding identification and management of stroke-related OPD. Interdisciplinary collaboration between nurses and speech–language therapists may improve nurses’ knowledge in identification and management of stroke-related OPD in lower middle-income settings such as South Africa.

## Introduction

In rural South Africa, strokes result in approximately 30 000 deaths yearly and are the largest cause of disability in the country (Maredza, Bertram, Gómez-Olivé, & Tollman, 2016). Stroke is one of the leading causes of oropharyngeal dysphagia (OPD) (Behera et al., 2018; Joundi et al., 2017). Oropharyngeal dysphagia is often underdiagnosed in people with stroke, leading to adverse complications including aspiration pneumonia, malnutrition, dehydration, extended hospital stay and death (Takizawa, Gemmell, Kenworthy, & Speyer, 2016).

Early identification of OPD using screening allows for prompt initiation of management plans, which may improve patient outcomes (Sherman et al., 2018).

Nursing staff are more likely to be the first health professionals to detect the early symptoms of OPD as they administer medication and feed patients (Bhimte & Rangasayee, 2015). People identified with swallowing difficulties should ideally be referred to a speech-language therapist (SLT) for comprehensive assessment and management (American Speech-Language-Hearing Association [ASHA], 2016). Only a limited number of SLTs are employed in the South African public sector, resulting in large caseloads (Ostrofsky & Seedat, 2016). Contextual challenges of limited resources, both human and physical, are a barrier and often result in long waiting times for the patient and possibly insufficient clinical management of dysphagia cases (Andrews & Pillay, 2017). The importance of accurate early identification of stroke-related OPD by nurses is thus evident, as this may guide its initial management, such as placing a patient *nil per os* until formal SLT review (Behera et al., 2018; Cummings et al., 2015; Palli et al., 2017). Oropharyngeal dysphagia management may include changes in consistencies, positioning or swallow manoeuvres (South African Speech-Language and Hearing Association [SASLHA], 2011).

Ideally, nurses should effectively identify OPD and risk for aspiration by screening patients’ swallowing abilities before administering food or liquids (Behera et al., 2018; Palli et al., 2017).

Nurses can implement initial swallowing precautions to prevent unfavourable outcomes until SLTs are able to perform comprehensive swallow assessments (Sherman et al., 2018). Patients who fail a screen and have possible OPD should be referred to an SLT for further assessment (Sherman et al., 2018). In lower-middle-income countries (LMICs) such as South Africa, swallow screenings by nurses are inconsistently conducted because of insufficient screening protocols and limited material and human resources (Blackwell & Littlejohns, 2010). The result is a lack of clarity as to who has the responsibility to refer to SLTs and which patients require swallowing assessment following stroke (Blackwell & Littlejohns, 2010).

In South Africa, healthcare professionals may not have a large amount of experience or training in stroke-related OPD (Willie, 2011). A recent unpublished master’s study stated that there is also a shortage of nurses in the public sector in South Africa, reducing the amount of time nurses may spend with patients (George, Gow, & Bachoo, 2013; Robbertse, 2018). Only 50% of nurses service the majority of patients in an under-resourced setting (Blackwell & Littlejohns, 2010). Nurses who receive sufficient training and support to perform dysphagia screenings are more likely to use swallow screening protocols and appropriately refer to SLTs (Sivertsen, Graverholt, & Espehaug, 2017). Yet international studies report that nurses display difficulty identifying OPD timeously following a stroke, delaying appropriate referral for intervention (Bhimte & Rangasayee, 2015).

Nursing staff in South Africa demonstrated low awareness of the role of SLTs in OPD management, possibly because of insufficient undergraduate training on OPD and minimal interaction with SLTs in practice (Albini, Soares, Wolf, & Gonҫalves, 2013; Robbertse, 2018).

Prevention of dysphagia-related complications and establishment of screening protocols to clearly indicate the roles and responsibilities of various professionals in a dysphagia team is the role of SLTs (ASHA, 2016). The SLT’s involvement in interdisciplinary teamwork and collaboration with nurses could ensure that team members are informed and adequately prepared for identification of OPD (Rhoda & Pickel-Voight, 2015). More regular interaction between nurses and SLTs can allow for sharing of knowledge and expertise between professions (Robbertse, 2018), which is essential within LMICs (Blackwell & Littlejohns, 2010). Improved interdisciplinary collaboration may lead to shorter hospital stays, lessen the number of patient complications and decrease mortality rates in people with stroke-related OPD (Dondorf, Fabus, & Ghassemi, 2015). The aim of this study was to describe nurses’ knowledge of stroke-related OPD in the Eastern Cape, South Africa which may identify possible training needs and guide future interprofessional collaboration between SLTs and nurses in this setting.

## Methods

### Study design and setting

Data were collected using a validated survey on eating and swallowing difficulties(Rhoda & Pickel-Voight, 2015) and a quantitative descriptive study design (Leedy & Ormrod, 2014). The survey was distributed by hand to 135 nurses from five sites in a metropolitan area of the Eastern Cape, South Africa. The study aimed to gather data in three areas: the identification, complications and management of stroke-related OPD.

### Study population and sampling

Participants were required to be qualified nurses working with people with stroke, who were proficient in English. Using purposive sampling, 135 participants were recruited (Leedy & Ormrod, 2014). Three surveys were returned incomplete, and two surveys were not returned; therefore, the responses of 130 participants were analysed. Participant characteristics are presented in Table 1. Approximately half the participants (*n* = 66; 50.8%) had 10 or more years of experience, and 86 participants (66.2%) were working in medical wards (Table 1).

**TABLE 1:** Participants’ socio-demographic characteristics and professional experience (*N* = 130).

Characteristic	*n*	%
Level of healthcare		
Primary	44	33.8
Secondary	50	38.5
Tertiary	36	27.7
Age (years)		
20–30	21	16.2
31–40	28	21.5
41–50	39	30.0
51–60	41	31.5
Over 60	1	0.8
Qualification		
Enrolled nursing assistant	29	22.3
Staff nurse	21	16.2
Professional nurse	80	61.5
Years of nursing experience		
0–3	17	13.0
4–6	27	20.8
7–9	20	15.4
10+	66	50.8
Current work context†		
Medical ward	86	66.2
Casualty	7	5.4
Out-patient clinic	20	15.4
Day hospital	24	18.5

† , Participants may be actively working in more than one context.

### Data collection

A published survey about eating and swallowing difficulties (Rhoda & Pickel-Voight, 2015) was used to collect data. The survey was previously validated by five practising SLTs with experience in assessment and management of OPD, and the test–retest method was used to ensure reliability (Rhoda & Pickel-Voight, 2015). The survey consisted of four sections: demographic information and level of experience, signs and symptoms, complications and management of dysphagia. A pilot study was conducted with 10 participants prior to data collection to ensure clarity of questions and increasing reliability of the findings. Data were collected from July to September 2018 by the first author, who distributed the hard copy surveys by hand and was available for clarification. Nurses who did not wish to participate were able to refuse participation from the outset or withhold submission of the survey.

### Data analysis

The survey was scored using the guidelines of Rhoda and Pickel-Voight (2015), tallying the number of correct responses in each section. Each participant’s level of knowledge was classified as follows: A score of ≥ 75% was ‘high’, 50% – 74% was ‘moderate’ and a score of ≤ 50% was ‘low’ (Rhoda & Pickel-Voight, 2015). Data were analysed using the Statistical Package for the Social Sciences version 25, using descriptive and inferential statistics in consultation with a statistician. The Cronbach’s alpha coefficient equalled 0.707, demonstrating the reliability of the questionnaire (Goforth, 2015). *P*-values of <0.05 were considered statistically significant. Variability of responses was noted across nurses and within levels.

### Ethical considerations

Ethical clearance was obtained from Faculty of Humanities and Health Sciences, University of Pretoria (protocol number GW20180105HS). Permission was obtained from the required provincial Department of Health governing bodies, with voluntary participation and written informed consent from each participant.

## Results

### Level of experience

Most participants (*n* = 118; 90.8%) had cared for a patient who had had a stroke, whereas only 50% (*n* = 65) had received training on stroke patient care. The majority of participants (*n* = 100; 76.9%) had cared for stroke patients with swallowing difficulties, with only 26.9% (*n* = 35) having received training on OPD and its management. Interestingly, 81.5% (*n* = 106) of participants indicated that they were unsatisfied with their current knowledge of stroke-related OPD, and most participants (*n* = 125; 96.2%) indicated a desire for further training. Variability of responses was noted across participants and within the different levels of healthcare.

### Identification of signs and symptoms of oropharyngeal dysphagia

Table 2 shows a comparison of participants’ understanding of the typical symptoms of OPD. In Tables 2 to 4, the *p*-values labelled ‘P–S’ (primary- and secondary-level groups), ‘P–T’ (primary- and tertiary-level groups), and ‘S–T’ (secondary- and tertiary-level groups) indicate whether correct responses differed significantly between groups.

**TABLE 2:** Comparison of participants’ knowledge of signs and symptoms of oropharyngeal dysphagia (*N* = 130).

Signs/symptoms	Correct answer	Primary (*n* = 44)		Secondary (*n* = 50)		Tertiary (*n* = 36)		Fisher’s exact test (*p*)			Total	
		*n*	%	*n*	%	*n*	%	P–S	P–T	S–T	*n*	%
Coughing whilst eating	A	36	81.8	46	92.0	32	88.9	0.215	0.532	0.715	114	87.7
Skin irritations	D	30	68.2	33	66.0	20	55.6	1.000	0.258	0.373	83	63.8
Feeling of food getting stuck in throat	A	38	86.4	45	90.0	29	80.6	0.750	0.551	0.227	112	86.2
Choking on saliva during non-mealtimes	A	37	84.1	44	88.0	28	77.8	0.766	0.569	0.244	109	83.8
Poor movement of tongue	A	41	93.2	43	86.0	31	86.1	0.327	0.457	1.000	115	88.5
Food remains in mouth	A	36	81.8	47	94.0	33	91.7	0.106	0.329	0.691	116	89.2
Poor chewing	A	37	84.1	44	88.0	33	91.7	0.766	0.499	0.729	114	87.7
Patients always cough if they aspirate	D	3	6.8	3	6.0	1	2.8	1.000	0.623	0.637	7	5.4
Difficulty closing lips	A	37	84.1	33	66.0	14	38.9	0.059	0.000*	0.016*	84	64.6
Weight loss	A	32	72.7	33	66.0	21	58.3	0.510	0.236	0.504	86	66.2
Frequent throat clearing after swallowing	A	29	65.9	31	62.0	18	50.0	0.830	0.176	0.280	78	60.0
Hoarse voice	A	25	56.8	31	62.0	8	22.2	0.676	0.003*	0.000*	64	49.2
Chest pain	A	11	25.0	18	36.0	17	47.2	0.272	0.059	0.375	46	35.4

A, agree; D, disagree; P, primary; S, secondary; T, tertiary.

Note: Results indicate number (%) of correct responses.

* , Statistically significant difference between number of correct responses between the two groups.

As a group, participants performed best in the identification section, with a mean correct response score of 8.7/13, indicating that 66.7% of the symptoms were identified correctly. However, when asked whether patients always cough if they aspirate, only 5.4% (*n* = 7) of participants responded correctly. Two other symptoms also generated low scores, namely ‘hoarse voice’ (49.2%; *n* = 64) and ‘chest pain’ (35.4%; *n* = 46). More common symptoms such as ‘coughing whilst eating’ (*n* = 114; 87.7%), ‘poor movement of the tongue’ (*n* = 115; 88.5%) and ‘food remaining in the mouth’ (*n* = 116; 89.2%) were accurately identified by most participants.

Statistically, there were no significant differences across nurses working at the various levels of healthcare in this section (*p* = 0.066). However, for the item ‘difficulty closing lips’, the percentage of correct responses differed significantly, with primary-level nurses scoring a high correct response rate (84.1%) and tertiary-level nurses scoring much lower (38.9%; *p* = 0.000). There was also a significant difference for this item between secondary-level nurses, with the next highest score (66.0%), and tertiary-level nurses (38.9%; *p* = 0.016). Interestingly, primary-level nurses (56.8%) had significantly more correct responses to the item ‘hoarse voice’ when compared to the tertiary-level nurses (22.2%; *p* = 0.003). The same trend was seen for this item amongst secondary-level nurses (62.0%), who demonstrated significantly more correct responses than the tertiary-level nurses (22.2%; *p* = 0.000).

### Complications of oropharyngeal dysphagia

Participants scored the lowest in this section of the survey, with a mean correct score of 4.73/10, revealing that only 47.3% of complications were accurately identified (Table 3).

**TABLE 3:** Comparison of participants’ knowledge of complications of oropharyngeal dysphagia (*N* = 130).

Complications	Correct answer	Primary (*n* = 44)		Secondary (*n* = 50)		Tertiary (*n* = 36)		Fisher’s exact			Total (*n* = 130)	
		*n*	%	*n*	%	*n*	%	P-S	P–T	S–T	*n*	%
Increased mortality	A	31	70.5	29	58	11	30.6	0.282	0.001*	0.016*	71	54.6
Pneumonia	A	23	52.3	30	60	15	41.7	0.533	0.376	0.126	68	52.3
Anaphylactic shock	D	9	20.5	12	24	11	30.6	0.805	0.314	0.622	32	24.6
General weakness	A	38	86.4	41	82	22	61.1	0.588	0.018*	0.047*	101	77.7
Problems with digestion	D	2	4.5	4	8	5	13.9	0.681	0.234	0.482	11	8.5
Aspiration	A	42	95.5	49	98	32	88.9	0.598	0.401	0.156	123	94.6
Dehydration	A	33	75	44	88	27	75	0.116	1.000	0.153	104	80
Sudden heart attack	D	7	15.9	15	30	14	38.9	0.144	0.013*	0.489	36	27.7
Malnutrition	A	36	81.8	39	78	30	83.3	0.798	1.000	0.594	105	80.8
Haematemesis (vomiting blood)	D	11	25	21	42	20	55.6	0.126	0.006*	0.275	52	40

Note: Results indicate the number (%) of correct responses.

* , Statistically significant difference between number of correct responses between the two groups: P = Primary, S = Secondary, T = Tertiary A, Agree; D, Disagree; P, Primary; S, Secondary; T, Tertiary.

Three complications were incorrectly thought to be the result of a stroke, with only a small percentage of participants identifying them correctly (Table 3). These were ‘anaphylactic shock’ (*n* = 32; 24.6%), ‘problems with digestion’ (*n* = 11; 8.5%) and ‘sudden heart attack’ (*n* = 36; 27.7%). Professional nurses performed better in this section when compared to enrolled nursing assistants (*p* = 0.015) and to staff nurses (*p* = 0.020). Table 3 shows that participants were not familiar with OPD complications that could arise following stroke. For the item ‘increased mortality’, primary-level nurses demonstrated the best knowledge (70.5%) when compared to tertiary-level nurses (30.6%; *p* = 0.001). However, the tertiary-level nurses (58.0%) also scored significantly lower on this item compared to the secondary-level nurses (*p* = 0.016). A similar result was found for ‘general weakness’, where both primary-level (86.4%) and secondary-level (82.0%) nurses had high scores, demonstrating better knowledge than the tertiary-level nurses (61.1%). The correct response rate for ‘sudden heart attack’ was low across groups, with the tertiary-level nurses having the highest score (38.9%) and primary-level the lowest (15.9%). For ‘haematemesis’, the tertiary-level nurses had the best knowledge (55.6%), and the primary-level nurses had the fewest correct responses (25.0%).

### Management practices for oropharyngeal dysphagia

Participants were required to determine whether or not certain management practices were appropriate (Table 4). Participants had a mean correct score of 3.8/7, reflecting an average of 54.2% for this section, indicating some difficulty identifying ideal management practices for patients with stroke-related OPD.

**TABLE 4:** Comparison of participants’ knowledge of the management of OPD (*N* = 130).

Management practices	Correct answer	Primary (*n* = 44)		Secondary (*n* = 50)		Tertiary (*n* = 36)		Fisher’s exact *p*-value			Total (*n* = 130)	
		*n*	%	*n*	%	*n*	%	P–S	P–T	S–T	*n*	%
Patients with a nasogastric tube need daily oral hygiene (mouth washing and brushing of the teeth)	A	42	95.5	48	96	33	91.7	1.000	0.653	0.645	123	94.6
Thickened liquid should be avoided	D	6	13.6	21	42	5	13.9	0.003*	1.000	0.008*	32	24.6
Watery liquids are the safest to drink	D	2	4.5	17	34	11	30.6	0.001*	0.002*	0.818	30	23.1
All patients with difficulty swallowing need a feeding tube	D	19	43.2	17	34	5	13.9	0.4	0.007*	0.046*	41	31.5
The best position while feeding the patient is when the patient lies flat on his back	D	37	84.1	46	92	31	86.1	0.337	1.000	0.482	114	87.7
The patient can always eat normal hospital food	D	37	84.1	40	80	22	61.1	0.789	0.024*	0.087	99	76.2
A feeding tube is only indicated in a patient with impaired consciousness	D	16	36.4	23	46	15	41.7	0.404	0.652	0.826	54	41.5

Note: Results indicate the number (%) of correct responses.

* , Statistically significant difference between number of correct responses between the two groups: A, Agree; D, Disagree; P, Primary; S, Secondary; T, Tertiary.

A few misconceptions were evident, indicated by low percentages of correct responses of disagreement with inappropriate management practices. These included that ‘thickened liquids should be avoided’ (*n* = 32; 24.6%), ‘watery liquids are the safest to drink’ (*n* = 30; 23.1%) and ‘all patients with difficulty swallowing require a feeding tube’ (*n* = 41; 31.5%). Remarkably, the secondary-level nurses had better knowledge on the item ‘thickened liquid should be avoided’ (42.0%) when compared to both primary-level (13.6%) and tertiary-level (13.9%) nurses. For ‘watery liquids are the safest to drink’, the secondary-level (34.0%) and tertiary-level (30.6%) nurses scored similarly, whereas the primary-level nurses had a low score (4.5%). On the item ‘all patients with difficulty swallowing need a feeding tube’, the primary-level nurses scored the highest (43.2%), followed by the secondary-level nurses (34.0%), and the tertiary-level nurses scored the lowest (13.9%).

Finally, a significant difference was found for ‘the patient can always eat normal hospital food’ between the primary (84.1%) and tertiary (61.1%) levels (*p* = 0.024).

Statistically, for the management of OPD section, the secondary-level nurses showed significantly better knowledge when compared to the primary-level nurses (*p* = 0.022) and the tertiary-level nurses (*p* = 0.010). The same is seen in the overall number of correct responses, with the secondary-level nurses displaying the best knowledge on OPD, when compared to the primary-level nurses (S–P: *p* = 0.044) and the tertiary level nurses (S–T: *p* = 0.025). The study revealed moderate scores overall across all levels of healthcare with a mean correct score of 17.20/30 (57.33%).

## Discussion

Despite regularly managing stroke patients, this study revealed that only half of the participants had received previous training on stroke, and only 26.9% of the participants had received training on stroke-related OPD.

Although research shows that OPD occurs in up to 80% of persons who have suffered a stroke (Takizawa et al., 2016), the nurses in this study, who are vital in the management of OPD, had received minimal training.

In order to manage OPD, it is important for nurses to have good knowledge of OPD symptoms for early identification and avoidance of unfavourable outcomes such as malnutrition, aspiration pneumonia and death (Behera et al., 2018; Palli et al., 2017). The results revealed that all three levels of nurses had better knowledge of the symptoms than about the complications and management of OPD. Interestingly, tertiary-level participants had the lowest scores for most items in this section, which may be attributed to the management of stroke patients in the South African healthcare system: Patients first present with a stroke at local clinics and are referred up the levels of healthcare as necessary; once medically stable, patients are often transferred back to a lower healthcare level for chronic management (KwaZulu-Natal Department of Health, 2014). Therefore, tertiary- and secondary-level nurses often only manage acute stroke patients with OPD.

Despite good knowledge of symptoms as a group, there was a misconception amongst participants on coughing as a sign of aspiration. Patients do not always cough when they aspirate (Miles et al., 2013); however, 83.1% (*n* = 108) of participants believed coughing was always present, indicating poor awareness of silent aspiration, a well-known symptom of stroke-related OPD (Felix, Joseph, & Daniels, 2019). Similar results were found by Rhoda and Pickel-Voight (2015), with 80.4% of nurses believing that coughing was always present during aspiration. If unidentified, silent aspiration places patients at risk for aspiration pneumonia (Teuschl et al., 2018). Therefore, it is essential for nurses across all levels of healthcare to be aware of all symptoms of OPD to effectively manage patients.

Only 52.3% of participants believed that pneumonia was a complication of OPD. A recent study indicated that stroke patients diagnosed with OPD are 4.69 times more likely to get aspiration pneumonia than those without OPD (Feng et al., 2019). For this reason, it is vital that nurses be aware of complications associated with mismanaged OPD and have knowledge of the importance of appropriate management. The participants had a mean correct score of 4.73/10 for identifying the complications associated with stroke-related OPD, indicating that nurses across all levels of healthcare in this study were unaware of adverse consequences resulting from undiagnosed or mismanaged OPD. The responses varied widely between items as well as between healthcare levels, which may result from the varying caseloads that nurses face. Similarly, primary-level nurses were the most accurate in identifying both increased mortality (70.5%) and general weakness (86.4%), followed by secondary-level (58.0% and 82.0%, respectively) and tertiary-level nurses with the lowest scores (30.6% and 61.1%, respectively) for these complications.

Most participants (*n* = 89; 68.5%) believed that all patients with OPD should be fed using nasogastric tubes, which may be because it is viewed as the predominant management option for stroke-related OPD. Secondary-level nurses had the best knowledge of the correct management procedures for patients with stroke-related OPD.

Overall, secondary-level nurses had the highest scores (P = 17.80/30; S = 18.94/30; T = 16.50/30) across all sections of the survey and therefore had the best knowledge of stroke-related OPD.

Nursing staff will most often be feeding or providing medication to persons with stroke; thus, knowledge of the delivery method of food and liquids, food consistency and positioning of patient is crucial (Bhimte & Rangasayee, 2015). Most participants (75.4%) were unaware of the value of thickening agents in the treatment of OPD. Thickening agents do not improve swallow physiology, but research emphasises the use of thickeners as a compensatory measure to improve swallow safety and reduce aspiration risk by slowing the flow of the bolus (Cichero et al., 2017).

A South African study conducted in two provinces found that nurses identified limited staff and time, as well as insufficient knowledge and training on OPD, as barriers to appropriate OPD management (Robbertse, 2018). The same can be seen in the current study, where the inconsistencies and misconceptions about OPD demonstrate a need for more knowledge and training. Similar results regarding knowledge and training across all sections of the survey were found by Rhoda and Pickel-Voight (2015). Nurses in the current study, as well as the study by Rhoda and Pickel-Voight (2015), demonstrated the best knowledge in the signs and symptoms of stroke-related OPD section of the survey. The previous study found a score of 64.62%, compared to 66.7% in this study. In contrast to the previous study, where the next best outcome was of the complications associated with stroke-related OPD (58.15%), nurses in this research had the fewest correct responses (47.3%).

Lastly, nurses in the previous study scored 49.28% for management of stroke-related OPD, in comparison to 54.2% in the current study.

According to the classification of scores, both studies revealed that nurses had moderate knowledge of stroke-related OPD, with previous research reporting a score of 57.34% and this study reporting 57.33%. This finding may indicate a need for improved training and opportunities for continued collaboration between SLTs and nurses for improving management of stroke-related OPD so as to optimise patient outcomes. It is essential to develop effective training opportunities on OPD in multiple modes and facilitate regular interaction between nurses and SLTs to improve the quality of patient care (Robbertse, 2018). Collaboration between SLTs and nurses, combining nurses’ experience with SLTs’ knowledge and expertise on stroke-related OPD, can be used to maximise health resources in a constrained environment in order to improve the interdisciplinary management of stroke patients with OPD in LMICs (Dondorf et al., 2015).

### Limitations and future recommendations

The research was conducted in only one municipality in the Eastern Cape, South Africa comprising three levels of healthcare. As there is limited research available in the local context regarding nurses’ knowledge and needs relating to OPD, reference was made to, and comparisons drawn from, an unpublished master’s study, given its relevance to this study. The data were collected in a metropolitan area, further limiting the generalisability of the findings. Larger-scale studies of a similar nature across several provinces are recommended. Further investigation may be done on the availability of training programs for nursing professionals, and support offered in hospitals regarding decisions for patients with stroke-related OPD. Research should aim to identify existing resources to implement better stroke-related OPD identification and management procedures in hospitals.

## Conclusion

The study revealed that secondary-level nurses had better knowledge of symptoms and management of stroke-related OPD than primary- or tertiary-level nurses. The findings highlighted nurses’ misconceptions about the possible complications associated with stroke-related OPD. Speech-language therapists need to assist in the development and implementation of sustainable protocols for OPD screening and management within resource-constrained environments such as the South African health sector.

Integrated work relations with an emphasis on collaboration between nurses and SLTs will optimise the use of limited health resources, promote patient safety and improve outcomes (Dondorf et al., 2015).
